# Supplementary material for: Bullying among nursing university students: Prevalence, characteristics, and public health implications
Source: PLOS Glob Public Health. 2026 Jan 9;6(1):e0005814. doi: 10.1371/journal.pgph.0005814 (PMC12788639; doi:10.1371/journal.pgph.0005814)
Supplement: S1 Table — (DOCX) [file pgph.0005814.s001.docx]

**S1 Table. Sociodemographic characteristics of nursing university students.**

| **Variables** | **n (240)** | **%** |
| --- | --- | --- |
| **Age (n=240)**  17-19  20-22  23-25  ≥26 | 68  106  46  20 | 28.3  44.2  19.2  8.3 |
| **Gender (n=240)**  Male  Female | 62  178 | 25.8  74.2 |
| **Where do you live? (n=240)**  Alone  With roommates / In a flat  With your own family | 19  130  91 | 7.9  54.2  37.9 |
| **Marital status (n=239)**  Single  Married | 220  19 | 92.1  7.9 |
| **Study phase (n=238)**  Early  Late | 95  143 | 39.9  60.1 |
| **Place of original residence (n=240)**  Urban  Non-Urban | 102  138 | 42.5  57.5 |
| **Father's educational level (n=240)**  Pre-University education  Undergraduate  Postgraduate | 165  50  25 | 68.8  20.8  10.4 |
| **Mother's educational level (n=240)**  Pre-University education  Undergraduate  Postgraduate | 202  30  8 | 84.2  12.5  3.3 |
| **Family economic status – monthly income in OMR (n=240)**  Low (<500)  Middle (500-1500)  High (>1500) | 47  146  47 | 19.6  60.8  19.6 |
| **How would you rate your general health? (n=240)**  Poor / Fair  Good  Very good  Excellent | 8  29  114  89 | 3.3  12.1  47.5  37.1 |
| **How often do you use the internet? (n=240)**  Once a day / others  Multiple Times a Day  **What methods do you usually use to access the internet?**  Wi-Fi connection at SQU campus  Wi-Fi connection at Home  Personal mobile data  Wi-Fi hotspots from friends / family  Wi-Fi hotspots in public places | 5  235  226  201  187  70  42 | 2.1  97.9  94.2  83.8  77.9  29.2  17.5 |
