# Supplementary material for: Bullying among nursing university students: Prevalence, characteristics, and public health implications
Source: PLOS Glob Public Health. 2026 Jan 9;6(1):e0005814. doi: 10.1371/journal.pgph.0005814 (PMC12788639; doi:10.1371/journal.pgph.0005814)
Supplement: S2 Table — (DOCX) [file pgph.0005814.s002.docx]

**S2 Table. Bullying experiences and related factors among nursing university students who reported being bullied.**

| **Variables** | **n (63)** | **%** |
| --- | --- | --- |
| **How often do you get bullied at SQU? (n=63)**  Daily  Weekly  Monthly  Once a year  A few times during the academic term | 4  7  6  12  34 | 6.3  11.1  9.5  19.0  54.0 |
| **Who has bullied you? (n=63)**  Roommates  SQU mates  Hospital staff  Administrative staff  Teachers/ instructors | 20  59  11  6  27 | 31.7  93.7  17.5  9.5  42.9 |
| **What type of bullying have you faced? (n=63)**  Sexual  Cyber  Physical  Mental or emotional  Verbal | 2  11  4  23  62 | 3.2  17.5  6.3  36.5  98.4 |
| **What are the negative effects of bullying on you? (n=63)**  Fearfulness  Feeling low  Poor marks  Change in appetite  Disengagement  Lack of motivation  Difficulty concentrating in class  Depression  Hate and anger  Nothing | 12  20  13  9  27  20  17  21  15  9 | 5.0  31.7  20.6  14.3  42.9  31.7  27.0  33.3  23.8  14.3 |
| **Which of the following methods have been used to bully you? (n=63)**  Online video clips of them  Online video clips of you  Chatroom  Through friends  Face to face messaging  Picture messages | 7  1  10  27  52  9 | 11.1  1.6  15.9  42.9  82.5  14.3 |
| **Where does bullying take place? (n=63)**  Sports  Corridors  Laboratory sessions  Resting rooms  Cafeteria/coffee  Other transport  Cars  In the buses  Hospital  Class rooms | 7  28  12  26  23  12  12  12  24  46 | 11.1  44.4  19.0  41.3  36.5  19.0  19.0  19.0  38.1  73.0 |
| **What do think makes them bully you? (n=63)**  Academic excellence  Poor academic performance  Exclusion from activities  Appearance or personal traits  Personal differences  Due to your academic major | 17  15  5  37  32  29 | 27.0  23.8  7.9  58.7  50.8  46.0 |
| **Have you complained about bullying? (n=63)**  Yes  No  If no, reasons for not complaining  Not sure to whom to complain  Fearfulness  Hope it will stop itself  Not important | 4  59  8  4  19  47 | 6.3  93.7  12.7  6.3  30.2  74.6 |
