# Supplementary material for: Bullying among nursing university students: Prevalence, characteristics, and public health implications
Source: PLOS Glob Public Health. 2026 Jan 9;6(1):e0005814. doi: 10.1371/journal.pgph.0005814 (PMC12788639; doi:10.1371/journal.pgph.0005814)
Supplement: Attachment — Submitted filename: Review Report PGPH-D-25-01719.docx [file pgph.0005814.s003.docx]

**Review Report**

**Manuscript Number**:	PGPH-D-25-01719

**Full Title:**	Bullying among nursing university students: prevalence, characteristics, and

public health implications

**General Comment:**

This study addresses an important issue surrounding bullying in nursing students. The topic is important and timely, but the author needs to revise the manuscript to clarify content and increase methodological rigor, and avoid disjointedness with the review of the literature, method, results, and finally conclusion. The author should address the comments below to enhance the quality and contribution of the study.

| **Article section** | **Comments/Evaluation** | |
| --- | --- | --- |
| **Participants**  **Line 140-141** | | Ensuring the accuracy and quality of the collected data should be included in the collection of data or data collection, not in this section. |
| **Participants**  **Line 141-143** | | It would be redundant to include this because you already mention the confidentiality in the Ethical Consideration section, and also the data-clearing process should be in the data collection section. Focus only in your participants, the criteria selection. |
| **Sample Size Collection** | | It would be better to provide the source or reference for the chosen sampling calculation. Citing an appropriate methodological reference will help justify that the computation used is valid and suitable for the study design |
| **Pilot Testing and Reliability** | | -It would be more appropriate if the participants in the pilot testing were not nursing students, since the actual participants of the study are nursing students. This would help avoid bias and strengthen the validity of the pilot results.  -Kindly provide a reference to support the claim that results slightly below the threshold are still acceptable because of sample size, item diversity, and cultural interpretation. |
| **Data Collection** | | The authors should provide additional details about how they ensured that the actual target participants—nursing students—were used to complete the online questionnaire using Google Forms. If verification measures were not made clear, then it is possible that responses were not obtained from only the intended target population. |
| **Questionnaire Structure** | | -Start with what kind of questionnaire you used, if it is researcher-made, the structure.  -Since the study utilized an online platform (Google Forms), the authors are requested to clarify how these consistent instructions were introduced (e.g., embedded in the form, via email, or through another channel) and how periodic reminders were delivered to participants |
| **Data Analysis** | | -This section should tell the reader *what you did with the data, how you did it, and why those methods were appropriate.* |
| **Results** | | The tables should be introduced in the text before presenting detailed discussions of the findings. Some results are explained without first referring to the corresponding tables, which may confuse readers. The authors are encouraged to introduce each table briefly (e.g., ‘Table 1 presents…’) and then proceed with the interpretation and discussion of the data |
| **Line 379-385** | | -The limitations of the study should not be placed in the Discussion section. It is more appropriate to create a separate section titled Limitations of the Study (or integrate it before the Conclusion), so that the Discussion remains focused on interpreting the findings in relation to the objectives and literature |
| **Redundancy**  **Line 285**  **Then Line 374** | | -*Nursing students who had experienced bullying at school or at home were significantly less likely*  The authors are advised to avoid repetition by consolidating the information in one place, ensuring that each result is stated once and elaborated upon in the appropriate section |
| **Results and Discussions** | | Kindly make sure that the Discussion matches the order of the Results. For instance, since the Results start with ‘Sociodemographic characteristics of nursing university students,’ this should also be the first topic explained in the Discussion. This parallel structure enhances coherence, clarity, and logical flow for the reader |
|  | | The inclusion of internet/Wi-Fi use in the study is noted, but the role and significance of this variable in relation to bullying are not clearly explained. The authors are encouraged to elaborate on how internet usage contributes to bullying experiences (e.g., as a medium for cyberbullying) and why it is important to consider in this context. Emphasizing this point would strengthen the rationale and highlight the relevance of addressing both traditional and online forms of bullying among nursing students |
| **Nothing else. Thank you and congratulations!** | | |
