# Supplementary material for: Bullying among nursing university students: Prevalence, characteristics, and public health implications
Source: PLOS Glob Public Health. 2026 Jan 9;6(1):e0005814. doi: 10.1371/journal.pgph.0005814 (PMC12788639; doi:10.1371/journal.pgph.0005814)
Supplement: Attachment — Submitted filename: Response to Reviewers.docx [file pgph.0005814.s005.docx]

**Rebuttal letter**

**We would like to thank the editor and reviewers for their invaluable feedback, insightful comments, and the considerable time they dedicated to reviewing our paper.**

**Below, we provide our detailed, point-by-point responses in bold to each of the reviewers' queries.**

Journal Requirements:

1. We have noticed that you have uploaded Supporting Information files, but you have not included a list of legends. Please add a full list of legends for your Supporting Information files after the references list.

**Response**

**The list of legends for Supporting Information files has been added.**

Reviewer #1: The study addresses an important issue surrounding bullying in nursing students. The topic is important and timely, but the author needs to revise the manuscript to clarify content and increase methodological rigor, and avoid disjointedness with the review of the literature, method, results, and finally conclusion. The author should address the comments to enhance the quality and contribution of the study.

| **Article section** | **Comments/Evaluation** | **Response** |
| --- | --- | --- |
| **Participants**  **Line 140-141** | Ensuring the accuracy and quality of the collected data should be included in the collection of data or data collection, not in this section. | **As suggested, we have moved the description of measures taken to ensure the accuracy and quality of the collected data from the Participants section to the Data Collection section.** |
| **Participants**  **Line 141-143** | It would be redundant to include this because you already mention the confidentiality in the Ethical Consideration section, and also the data-clearing process should be in the data collection section. Focus only in your participants, the criteria selection. | **As suggested, we have revised the Participants section to focus solely on participant characteristics and selection criteria. Details related to data accuracy, cleaning, and instructions provided to participants have been moved to the Data Collection section. Confidentiality is addressed in the Ethical Considerations section to avoid redundancy.** |
| **Sample Size Collection** | It would be better to provide the source or reference for the chosen sampling calculation. Citing an appropriate methodological reference will help justify that the computation used is valid and suitable for the study design | **As recommended, we have added an appropriate methodological reference to support the sampling calculation and to justify its suitability for the study design.** |
| **Pilot Testing and Reliability** | -It would be more appropriate if the participants in the pilot testing were not nursing students, since the actual participants of the study are nursing students. This would help avoid bias and strengthen the validity of the pilot results.  -Kindly provide a reference to support the claim that results slightly below the threshold are still acceptable because of sample size, item diversity, and cultural interpretation. | **We acknowledge that using nursing students for the pilot may have introduced overlap with the target population; however, this choice was made for feasibility and to ensure clarity and cultural relevance of the questionnaire. This limitation is now noted in the manuscript. Regarding Cronbach’s alpha, the value of 0.68 is slightly below the 0.70 threshold but still acceptable in exploratory studies, particularly with diverse items or cultural adaptations, and references have been added.** |
| **Data Collection** | The authors should provide additional details about how they ensured that the actual target participants—nursing students—were used to complete the online questionnaire using Google Forms. If verification measures were not made clear, then it is possible that responses were not obtained from only the intended target population. | **Details have been added to the Data Collection sections clarifying that the survey was distributed only through official institutional emails and verified College of Nursing WhatsApp groups. In addition, participants were required to confirm their enrollment in the Nursing program in the demographics section to ensure responses were limited to nursing students.** |
| **Questionnaire Structure** | -Start with what kind of questionnaire you used, if it is researcher-made, the structure.  -Since the study utilized an online platform (Google Forms), the authors are requested to clarify how these consistent instructions were introduced (e.g., embedded in the form, via email, or through another channel) and how periodic reminders were delivered to participants | **The questionnaire was researcher-developed and structured into four sections, with several items adapted from previously validated instruments, as now clarified at the beginning of the Questionnaire Structure subsection. We have also elaborated on the administration process: consistent instructions were embedded directly within the Google Forms platform to ensure all participants received the same guidance before beginning the survey. In addition, periodic reminders were sent to students through official institutional emails and verified College of Nursing WhatsApp groups during the data collection phase. These clarifications have been added to the revised manuscript** |
| **Data Analysis** | -This section should tell the reader *what you did with the data, how you did it, and why those methods were appropriate.* | **The Data Analysis section has been expanded to clarify not only what statistical methods were applied, but also why they were appropriate for the study objectives. Categorical variables were summarized using frequencies and percentages to describe the sample. The Chi-square test was used to examine associations between categorical variables, as it is suitable for detecting group differences. Binary logistic regression was employed to identify independent predictors of bullying, as the outcome variable was dichotomous. Variables with a crude p-value less than 0.25 in the bivariate analysis were entered into the regression model to avoid omitting potential confounders. A significance level of p < 0.05 was adopted to determine statistical significance.** |
| **Results** | The tables should be introduced in the text before presenting detailed discussions of the findings. Some results are explained without first referring to the corresponding tables, which may confuse readers. The authors are encouraged to introduce each table briefly (e.g., ‘Table 1 presents…’) and then proceed with the interpretation and discussion of the data | **As suggested, we have revised the Results section to ensure that each table is introduced in the text before detailed findings are discussed.** |
| **Line 379-385** | -The limitations of the study should not be placed in the Discussion section. It is more appropriate to create a separate section titled Limitations of the Study (or integrate it before the Conclusion), so that the Discussion remains focused on interpreting the findings in relation to the objectives and literature | **In line with the suggestion, we have moved the limitations into a separate section titled** **Limitations of the Study, placed before the Conclusion.** |
| **Redundancy**  **Line 285**  **Then Line 374** | -*Nursing students who had experienced bullying at school or at home were significantly less likely*  The authors are advised to avoid repetition by consolidating the information in one place, ensuring that each result is stated once and elaborated upon in the appropriate section | **To address the redundancy, we removed the repeated sentences from the Discussion section. This result is now stated once and elaborated upon in the Discussion section to avoid repetition and improve clarity.** |
| **Results and Discussions** | Kindly make sure that the Discussion matches the order of the Results. For instance, since the Results start with ‘Sociodemographic characteristics of nursing university students,’ this should also be the first topic explained in the Discussion. This parallel structure enhances coherence, clarity, and logical flow for the reader | **We thank the reviewer for this useful comment. While the Results section begins with the sociodemographic characteristics of the participants, these variables were mainly presented to describe the sample and were not central to the study objectives. For this reason, we did not include a separate discussion of these characteristics.** |
|  | The inclusion of internet/Wi-Fi use in the study is noted, but the role and significance of this variable in relation to bullying are not clearly explained. The authors are encouraged to elaborate on how internet usage contributes to bullying experiences (e.g., as a medium for cyberbullying) and why it is important to consider in this context. Emphasizing this point would strengthen the rationale and highlight the relevance of addressing both traditional and online forms of bullying among nursing students | **As suggested, we have expanded the discussion section to clarify the relevance of internet and Wi-Fi use in relation to bullying. We have added this information: “Nearly all students reported frequent internet access, primarily through Wi-Fi at the university and at home. Notably, those who used the internet once a day or less were significantly less likely to report being bullied compared to frequent users (OR = 0.059, p = 0.012). This suggests that high levels of online activity may increase exposure to bullying behaviors, particularly through digital platforms, even though most reported bullying occurred face-to-face. The internet may therefore act as both a medium for cyberbullying and a facilitator of negative peer interactions. These findings underscore the importance of addressing both traditional and online forms of bullying when developing preventive strategies and health promotion programs in nursing education”.** |
| **Nothing else. Thank you and congratulations!** | | **Thank you** |

Reviewer #2: The article is well-written. However, several methodological and reporting issues need clarification and strengthening to make the article suitable for publication.

1. Sampling method:
The study used convenience sampling. This limits generalizability. The authors should justify why random or stratified methods were not possible. Possible selection bias (students more affected by bullying may have been more motivated to participate).

**Response**

**We acknowledge that the use of convenience sampling limits the generalizability of our findings. While random or stratified sampling methods were theoretically possible, they were not applied due to practical considerations. Convenience sampling was chosen to ensure feasibility within the study’s timeframe, to maximize participation during scheduled sessions, and to secure an adequate sample size. We agree with the reviewer that this approach may introduce selection bias, as students with stronger experiences of bullying may have been more motivated to participate. This point has now been added in the limitations section of the revised manuscript.**

2.Generalizability:
The study is conducted at a single university. Authors should emphasize that the results may not represent all nursing students in Oman or the Gulf region.

**Response**

**We agree that conducting the study at a single governmental university limits the representativeness of the findings. We have emphasized this point in the limitations section by clarifying that the results may not fully represent all nursing students in Oman or in the wider Gulf region.**

3.Measurement reliability:
Cronbach's alpha for the pilot questionnaire was 0.68, which is below the conventional 0.7 threshold. The authors acknowledge this, but they should discuss implications for the validity of findings.

**Response**

**We acknowledge that a Cronbach’s alpha of 0.68 falls slightly below the conventional 0.7 threshold. However, values between 0.6 and 0.7 are often considered acceptable in exploratory research, particularly in studies addressing complex social and behavioral constructs such as bullying, where heterogeneity of experiences may reduce internal consistency. We have now expanded the limitations sections to clarify that while the slightly lower reliability coefficient may indicate moderate internal consistency, it does not invalidate the findings. Rather, it highlights the need for cautious interpretation and suggests that future studies could further refine the instrument to strengthen reliability.**

4.Abstract: Overly detailed. Could be shortened by focusing on the most critical results and implications.

**Response**

**As recommended, we have revised the abstract to focus on the most critical results and implications, thereby improving clarity.**

5.Tables: Some tables contain large amounts of data that may be better presented in supplementary files

**Response**

**As suggested, we have moved Table 1 (detailed sociodemographic characteristics) and Table 3 (in-depth breakdown of bullying experiences among victims) to the supplementary files. The key summary information from these tables is retained in the text. Tables 2, 4, and 5, which present the most critical findings on prevalence, comparisons, and regression analyses, remain in the main manuscript for clarity.**

**All editor and reviewers’ comments have been carefully addressed in the revised manuscript. We greatly appreciate the editor and reviewers' evaluation and constructive feedback.**

**Thank you for your time and effort**s
