# Supplementary material for: Bullying among nursing university students: Prevalence, characteristics, and public health implications
Source: PLOS Glob Public Health. 2026 Jan 9;6(1):e0005814. doi: 10.1371/journal.pgph.0005814 (PMC12788639; doi:10.1371/journal.pgph.0005814)
Supplement: Attachment — Submitted filename: Response_to_Reviewers_auresp_2.docx [file pgph.0005814.s006.docx]

**Rebuttal letter**

**We would like to thank the editor and reviewers for their invaluable feedback.**

**Below, we provide our detailed, point-by-point responses in bold to each of the reviewers' queries.**

Guidelines for resubmitting your figure files are available below the reviewer comments at the end of this letter.

**Response**

**As suggested, both figures have been already revised and checked using the PLOS NAAS tool.**

Reviewer #1: The updated manuscript responds to all comments and suggestions made by the reviewers. Changes are made and specified clearly the rebuttal letter and in the main text, to clarify the methodology, increase coherence, and to strengthen the analysis.

**Response**

**Thank you**

Reviewer #2: The study design is appropriate for its exploratory aims, the statistical analyses (descriptive statistics, chi-square tests, and multivariate logistic regression) are sound, and the conclusions are supported by the data.

**Response**

**Thank you**

1-The abstract is now more focused, but the sentence on data analysis could be tightened for brevity

**Response**

**As suggested, the sentence on data analysis has been revised.**

2-Methods - Data Analysis (lines 165-174): The use of a crude p-value <0.25 for variable inclusion in multivariate models is justified to avoid overlooking confounders, but cite a reference for this approach (e.g., Hosmer and Lemeshow’s text on logistic regression) to support it.

**Response**

**As suggested, the following citation has been included in the methods section:** **Hosmer DW, Lemeshow S. Applied logistic regression. 2nd ed. New York: John Wiley & Sons; 2000. pp. 95–97.**

**Subsequently, other references have been rearranged.**

3. Results (lines 200-209): The detailed breakdown in S2 Table is useful, but in the text, clarify that the percentages for bullying effects (e.g., disengagement 42.9%) are among the 63 bullied students. This avoids any misinterpretation.

**Response**

**As suggested, the results section has been revised to clarify that the reported percentages for bullying effects (e.g., disengagement 42.9%) refer specifically to the subgroup of 63 students who reported being bullied.**

4-The integration of logistic regression findings with public health implications is strong. However, suggest practical recommendations, such as digital literacy training for early-phase students to mitigate internet-related risks.

**Response**

**As suggested, the discussion has been expanded to include practical recommendations based on our findings, particularly emphasizing the importance of digital literacy training and early-phase student support to mitigate internet-related risks.**

**We greatly appreciate the editor and reviewers' evaluation and constructive feedback.**

**Thank you for your time and effort**s
