# Supplementary material for: Bullying among nursing university students: Prevalence, characteristics, and public health implications
Source: PLOS Glob Public Health. 2026 Jan 9;6(1):e0005814. doi: 10.1371/journal.pgph.0005814 (PMC12788639; doi:10.1371/journal.pgph.0005814)
Supplement: Attachment — Submitted filename: Response_to_Reviewers_auresp_3.docx [file pgph.0005814.s007.docx]

**Rebuttal letter**

**We would like to further thank the editor and reviewers for their invaluable feedback.**

**Below, we provide our detailed, point-by-point responses in bold to each of the reviewers' queries.**

Guidelines for resubmitting your figure files are available below the reviewer comments at the end of this letter.

**Response**

**As suggested, both figures have been already revised and checked using the PLOS NAAS tool.**

Reviewer #1: I appreciate the authors taking all comments into account. All the concerns that were previously raised by the reviewer(s) have been addressed satisfactorily—the manuscript has improved tremendously; therefore, the manuscript is acceptable in its current state.

**Response**

**Thank you.**

Reviewer #2: The article is scientifically and content-wise ready and has no major problems.

There are only a few corrections in the format, references and consistency of the text that can be easily fixed.

1- Go through the final version of Word once and remove duplicates.

**Response**

**As suggested, we carefully reviewed the final version of the manuscript and removed all duplicate statements.**

2-In the abstract, you can add a stronger conclusion sentence.

**Response**

**As suggested, we have added a stronger conclusion sentence.**

3- Write at least one sentence about tables

**Response**

**As suggested, we have added an interpretive sentence describing the content and purpose of each table.**

**We greatly appreciate the editor and reviewers' evaluation and constructive feedback.**

**Thank you for your time and effort**s
